# The clinical characteristics of familial cluster headache

**DOI:** 10.1177/03331024221076478

**Published:** 2022-02-15

**Authors:** Emer O’Connor, Elham Nikram, Lou Grangeon, Daisuke Danno, Henry Houlden, Manjit Matharu

**Affiliations:** 1 Headache and Facial Pain Group, University College London (UCL), Queen Square Institute of Neurology and The National Hospital for Neurology and Neurosurgery, London, UK; 2 Department of Neuromuscular Diseases, Institute of Neurology, University College London, UK; 3 Peninsula Technology Assessment Group (PenTAG), University of Exeter, UK; 4 Department of Neurology, Rouen University Hospital, Rouen, France

**Keywords:** Cluster headache, genetic, family history, familial

## Abstract

**Background:**

A positive family history predisposes to the development of cluster headache. The distinct characteristics of familial cluster headache have yet to be confirmed, however, evidence suggests a younger age of onset and higher proportion of females in this subgroup.

**Objectives:**

To assess the rate and mode of inheritance of familial cluster headache in a tertiary referral centre for headache. To describe the clinical features of familial cluster headache.

**Methods:**

A retrospective study conducted between 2007 and 2017. Cluster headache was confirmed in probands and affected relatives. Differences in demographics, clinical characteristics, and response-to-treatment in familial cluster headache were delineated through multivariate analysis using a control cohort of 597 patients with sporadic cluster headache.

**Results:**

Familial cluster headache was confirmed in 48 (7.44%) patients and predominantly reflected an autosomal dominant mode of inheritance with reduced penetrance. Familial cases were more likely to report nasal blockage (OR 4.06, 95% CI; 2.600–6.494, p < 0.001) during an attack and a higher rate of concurrent short-lasting unilateral neuralgiform headache with conjunctival injection and tearing (OR 3.76, 95% CI; 1.572–9.953, p = 0.004).

**Conclusion:**

These findings add to evidence suggesting a genetic component to cluster headache. Here, we demonstrated prominent nasal blockage, and a higher occurrence of concomitant short-lasting unilateral neuralgiform headache with conjunctival injection and tearing in this subgroup, further delineating the phenotype.

## Introduction

The familial aggregation of cluster headache (CH) has been described across a number of populations (1). Cases of concordance amongst monozygotic twins have also been reported (2). The true prevalence of familial CH is disputed, with some epidemiological studies estimating it to be as low as 2.3% whilst others report it to be as high as 20% (3,4). A number of factors including population stratification and reporting methods may contribute to this discrepancy. The largest study, comprising of 1720 CH patients, identified a positive family history in 75 patients, just over 4% of their cohort (5). Recently, a systematic review of all published data reporting familial CH estimated a rate of 0–22%, with a median rate of 8.2% (6). This is slightly higher than a meta-analysis suggesting a rate of approximately 6.27% (7). Overall, the cumulative evidence indicates that the number of affected individuals within families considerably exceeds the estimated prevalence of CH occurring sporadically within the general population of approximately 0.1% (8).

A large proportion of reported cases of familial CH consist of small pedigrees, often with only two affected individuals. While these findings may represent a chance association, they likely point to the inheritance of a genetic risk for the development of CH. Unfortunately, complex inheritance patterns, poor penetrance, intra-familial clinical heterogeneity, and the existence of atypical phenotypes within families impede familial genetic studies (5). The majority of genetic studies to date focus on candidate genes. For example, the hypocretin receptor 2 gene (*HCRTR2*), involved in the orexinergic system, has previously shown an association (9). However, additional population-based studies have failed to reproduce these findings (10). Other plausible candidates have also demonstrated an association, including the *CLOCK* (Circadian Locomotor Output Cycles Kaput, OMIM: 601851) gene, *ADH4* (Alcohol Dehydrogenase 4, OMIM: 103740) gene, and the *ANO3* (Anoctamin 3, OMIM: 610110) gene (11–13). These findings have yet to be replicated. Recently, for the first time, replicable genetic susceptibility loci, on chromosomes 2 and 6, were identified in genome-wide association studies (GWAS) of CH, further implicating genetic variation in its aetiology (14).

The majority of familial CH cases exhibit an autosomal dominant mode of inheritance, however, cases of autosomal recessive inheritance are also described (15,16). Penetrance appears to be lower in women than in men, with prominent father-to-son transmission (5,17). This lack of consistency across pedigrees may indicate loci heterogeneity - the cumulative effect of more than one variant or the influence of genetic and environmental modifiers.

The phenotypes of familial and sporadic CH appear to have similar clinical features (18). An earlier age of onset has been observed in the relatives of sufferers (19). Sporadic CH shows a clear male preponderance compared to familial CH where the gender ratio appears lower (20). To date, no study has performed an in-depth interrogation of the precise clinical characteristics specific to familial CH.

The aims of this study were to estimate the occurrence of a family history in our cohort of CH patients and to identify the likely mode of inheritance in these families. Furthermore, to delineate the differing clinical parameters between patients with familial CH compared to those with the sporadic form.

## Materials and methods

A retrospective study was conducted in the Headache clinic at the National Hospital for Neurology and Neurosurgery (Queen Square, London, UK) between January 2007 and April 2017. All consecutive patients diagnosed with CH in accordance with The International Classification of Headache Disorders 3rd edition (ICHD-3) underwent a detailed family history as part of their clinical assessment (21). Those with a first- or second-degree relative affected with CH were included in the study. Pedigrees were recorded for all familial CH cases. A diagnosis of CH was verified in relatives by a neurologist through direct clinical examination or phone consultation using a semi-structured interview based on the ICHD-3 criteria. In cases where relatives were deceased, only those with a diagnosis of CH confirmed by a neurologist were included.

Clinical data collected focused on pertinent demographics including sex and age of onset, laterality, site and quality of pain, attack characteristics (frequency, duration, and severity), associated symptoms (including autonomic and migrainous features), concurrent headache syndromes (as defined by ICHD-3) and response to acute and prophylactic treatments (Supplementary table 1). Intractability to treatment was defined using the description by European Headache Federation and Goadsby et al. (22,23). An adequate response to preventive treatment was defined as a ≥50% reduction in attack frequency. For acute treatment, a ≥50% reduction in pain at least 50% of the time was deemed satisfactory (24) (Supplementary table 2).

All participants gave informed consent Ethics board approval was obtained from the National Hospital for Neurology and Neurosurgery Research Ethics Committee, London, UK (REC number: 07/Q0512/26)

### Statistical analysis

To identify differences in clinical characteristics, cases of familial CH were compared to those with sporadic CH. In this analysis, descriptive statistics were expressed as a mean with standard deviation (SD). Before starting the analysis, missing values were accounted for using imputation techniques based on random forests. Missing values were not deliberately omitted and occurred in the retrospective ascertainment of data where some details were occasionally unavailable at random. As the dataset was highly imbalanced, the ROSE algorithm was used to balance the data (25). The ROSE function creates an artificially balanced sample according to a smoothed bootstrap approach. Univariate and multivariate analysis was then performed using the ROSE-adjusted sample (Rose sample).

In the univariate analysis, a Mann-Whitney U nonparametric test was utilised for continuous data and a Fisher exact or Chi-squared test for categorical data. For multivariate analysis, the LASSO algorithm was used to select relevant explanatory variables as there are no clinical grounds to predict relevant variables (26). The LASSO performs automatic variable selection and has the capacity to select groups of correlated variables. A logistic regression model was then fitted with the variables selected by the LASSO. The analysis was performed using R; the random forest, ROSE and glmnet packages were used (27). The threshold for statistical significance was set to p ≤ 0.05.

## Data availability statement

De-identified database and statistical analysis plan will be shared upon reasonable request for two years after publication

## Results

A total of 645 patients were included in the study. Of these, 456 (70.7%) were male. A family history of CH was reported in 66 patients (10.2%), the remainder were categorised as sporadic CH and used as controls. Probands were excluded because their affected relative(s) were deceased and lacked an official diagnosis (n = 7), they did not fulfil the ICHD3β diagnostic criteria (n = 6), and they declined participation or were uncontactable (n = 5). Overall, forty-eight (7.4%) individuals had a confirmed family history of CH.

In the familial CH cohort, 27 patients had episodic CH and 21 had chronic CH. The mean age of cases was 48.9 years (SD 12.05) and the mean age of onset was 28.48 years (SD 13.09). The mean follow-up time at clinic was 7.08 years (SD3.8) years. The mean duration of attacks was 67.2 mins (SD 43.6) and the mean frequency of attacks per day was three (SD 2). Only one patient lacked autonomic symptoms but experienced restlessness. In terms of concomitant headache, 15 cases had concurrent migraine and three were diagnosed with a concomitant trigeminal autonomic cephalalgia (TAC), all of which had SUNCT (short-lasting unilateral neuralgiform headache with conjunctival injection and tearing).

Four affected family members were observed in three family pedigrees, 11 families had three affected individuals and the remaining families consisted of only two affected individuals. One set of concordant monozygotic twins with no other affected family members also featured in the cohort. The majority of the families included were small, consisting of only two or three generations, impeding pedigree analysis. However, the most common mode of inheritance observed was most consistent with autosomal dominant transmission, observed in 40 families. However, of these, nine families exhibited evidence of reduced penetrance. The remaining eight had a pattern potentially consistent with an autosomal recessive pattern of inheritance. Transmission from parent to child was the most frequent mode of inheritance. In one family, both parents of the proband suffered from CH. All pedigrees are available in Supplementary Figure 1.

### Univariate analysis

Univariate analysis of ROSE-adjusted dataset identified several significant variables relevant to the familial CH group (Table 1, Supplementary Table 3). These included a younger age of onset (27.53 years, SD 14.25) compared to the sporadic group (31.80 years, SD 14.27, p < 0.001). Attack duration was shorter in familial CH group (70.35 +/− 49.42 minutes versus 91.49 +/− 90.86 minutes, p < 0.001). Autonomic symptoms were more prominent in patients with a family history of CH including eyelid oedema (176 [56.23%] vs 128 [38.55%], p < 0.001), conjunctival injection (270 [86.26%] vs 247 [74.39%], p < 0.001), lacrimation (283 [90.41%] vs 281 [84.63%], p = 0.03), nasal blockage (278 [88.81%] vs 218 [65.66%] p < 0.001), facial sweating (201 [64.21%] vs 175 [52.71%], p = 0.003), or flushing (142 [45.36%] vs 123 [37.04%], p = 0.03). An occipital (42[13.41%] vs 82 [24.69%], p < 0.001) and frontal (86 [27.47%] vs 119 [35.84%], p = 0.02) location of pain was more frequently identified in the sporadic group and cheek pain was more common in the familial group (90 [28.75%] vs 68 [20.48%], p = 0.018). Concomitant SUNCT occurred more frequently in the familial group (22 [7.02%] vs 8 [2.40%], p = 0.009). In addition, a poor response to high-flow oxygen as a treatment was significant in those with a positive family history (response rate of 65 [20.76%] vs 248 [74.69%], p 0.002).

**Table 1.** Baseline demographics and clinical characteristics of cohorts, with corresponding univariate analysis following imputation and re-balancing of cohorts.

Cohorts	Demographics and Clinical Characteristics		Imputed and re-balanced cohorts & Univariate Analysis		
	Familial CH (n = 48)(%)	Sporadic CH (n = 597)(%)	Familial CH ROSE sample (n = 313)(%)	Sporadic CH ROSE sample (n = 332)(%)	P value
Age	48.91+/−12.05	49.49+/−12.46	47.93+/−12.91	49.94+/−13.56	0.09
Gender M:F	35:13	421:176	225:88	237:95	0.95
Age of onset	28.48+/−13.09	31.29+/−13.13	27.53+/−14.25	31.80+/−14.27	**<0.001**
Chronic	21 (43.75)	285 (47.73)	137 (43.76)	166 (50.00)	0.13
Site					
Orbital	33 (68.8)	419 (70.18)	228 (72.84)	227 (68.37)	0.24
Frontal	14 (29.1)	203 (34)	86 (27.47)	119 (35.84)	**0.02**
Temporal	22 (45.8)	304 (50.9)	147 (46.96)	171 (51.50)	0.28
Parietal	7 (14.5)	106 (17.7)	48 (15.33)	70 (21.08)	0.07
Occipital	9 (18.75)	122(20.43)	42 (13.41)	82 (24.69)	**<0.001**
Cheek	14 (29.2)	136 (22.8)	90 (28.75)	68 (20.48)	**0.018**
Teeth	4 (8.3)	59 (9.88)	33 (10.54)	42 (12.65)	0.47
Ear	4 (8.3)	59 (9.88)	24 (7.66)	35 (10.54)	0.25
Autonomics					
Absence of Autonomics	1 (2.1)	13 (2.2)	3 (0.95)	13 (3.91)	0.62
Ptosis	28 (58.3)	345 (57.8)	207 (66.13)	211 (63.55)	0.54
Eyelid oedema	21 (43.8)	213 (35.7)	176 (56.23)	128 (38.55)	**<0.001**
Conjunctival Injection	34 (70.8)	401 (67.2)	270 (86.26)	247 (74.39)	**<0.001**
Miosis	1 (2.1)	22 (3.7)	49 (15.65)	35 (10.54)	0.07
Lacrimation	39 (81.25)	472 (79.1)	283 (90.41)	281 (84.63)	**0.03**
Nasal blockage	36 (75)	350 (58.6)	278 (88.81)	218 (65.66)	**<0.001**
Rhinorrhoea	30 (62.5)	364 (60.9)	245 (78.27)	244 (73.49)	0.18
Facial Sweating	23 (47.9)	295 (49.4)	201 (64.21)	175 (52.71)	**0.003**
Flushing	19 (39.6)	231 (38.7)	142 (45.36)	123 (37.04)	**0.03**
Aural Fullness	8 (16.7)	105 (17.6)	88 (28.11)	71 (21.38)	0.06
Agitation	40 (68.9)	498 (83.4)	288 (92.01)	287 (86.44)	**0.03**
Frequency/Duration of attacks					
Average Attacks per day	3.08+/−2.18	2.99+/−2.18	3.11+/−2.56	2.96+/−2.29	0.50
Average Duration (mins)	67.22+/−43.56	94.97+/−132.36	70.35+/−49.42	91.49+/−90.86	**<0.001**
Associated Headaches					
Migraine	15 (31.3)	180 (30.2)	85 (27.15)	97 (29.21)	0.62
TACS	3 (6.25)	20 (3.3)	22 (7.02)	8 (2.40)	**<0.009**
Treatment Response					
Oxygen non-responders	9 (18.7)	84 (14.1)	65 (20.76)	248 (74.69)	**0.002**
Sumatriptan non-responders	6 (12.5)	29 (4.8)	85 (27.15)	80 (24.09)	0.42
Intractable to preventative treatment	24 (50)	244 (40.8)	227 (72.52)	217 (65.36)	0.06

Information on univariate comparisons on imputed data is provided in Supplementary Table 3. CH: cluster headache, M: male, F: female, sc: subcutaneous, TACS: trigeminal autonomic cephalalgia.

### Multivariate analysis

Among all the variables in the dataset, LASSO algorithm selected age of onset, frontal site, occipital site, eyelid oedema, conjunctival injection, miosis, lacrimation, nasal blockage, Attack duration, and concurrent trigeminal autonomic cephalalgias for the logistic regression model. The multivariate analysis in logistic regression model with the selected variables recognise following bundle of variable to have significant impact on the outcome: Age of onset, presence of nasal blockage, attack duration, associated SUNCT. Results of multivariate analysis are summarised in Table 2. Consistent with univariate analysis, we identified a significant association between prominence of nasal blockage and the familial subgroup (OR 4.06, 95% CI 2.600–6.494; p < 0.001). Concomitant SUNCT was associated with familial CH (OR 3.76, 95% CI; 1.572–9.953, p = 0.004). Correction for multivariate modelling produced an odds ratio close to one for age of onset (OR 0.98, 95% CI 0.971–0.996, p < 0.009) and attack duration (OR 0.997, 95% CI 0.994-0.999 p = 0.012). This is likely owing to the large SD for both familial CH and sporadic CH.

**Table 2.** Summary of significant variables identified on multivariate analysis.

Predictive factor	OR	95% CI	p value
Age of onset	0.98	0.971–0.996	0.009
Presence of nasal blockage	4.06	2.600–6.494	<0.001
Attack duration	0.997	0.994–0.999	0.012
Associated SUNCT	3.76	1.572–9.953	0.004

SUNCT: Short-lasting, unilateral, neuralgiform headache attacks with conjunctival injection and tearing.

## Discussion

This study identified a possible family history of CH in 10.2% of cases. Further evaluation of suspected affected relatives demonstrated a familial rate of 7.44%. A diagnosis of CH was incorrectly attributed by probands to five individuals who fulfilled the ICHD3β criteria for migraine. Similarly, one relative was excluded due to atypical features of CH precluding clinical confirmation. As CH is a clinical diagnosis, this disparity reflects the documented diagnostic challenges associated with headache disorders, often requiring specialist input (28). Furthermore, it highlights the weaknesses of a family history taken by proxy and the essential requirement for clinical validation to provide accurate rates of family history in headache epidemiological studies.

Our findings are similar to rates reported in several studies where a diagnosis of CH was confirmed in an affected relative and the cumulative estimates of 6.27–8.2% (6,7). This provides further evidence for the role of heredity in the aetiology of CH. The extent of this, and the influence of environmental factors on the development of disease, remains unclear. Previously, a relative risk as high as 45.6-fold was predicted for individuals with a first degree relative with CH (29). Other studies describe a much lower familial prevalence (17). These findings should be examined in the context of an overall increase in the prevalence of CH due to recent improvements in awareness of the condition and adherence to diagnostic criteria (30).

The presence of relatives with atypical CH within families, who perhaps reflect part of a clinical spectrum, also impede a precise evaluation of the rate of familial CH (20). Similar to our cohort, these cases are often omitted from epidemiological studies as they do not strictly fulfil diagnostic criterion, but perhaps carry the same genetic risk with alternate modifiers. In contrast, shared environmental risk factors may influence the development of the phenotype and therefore contribute to familial clustering. Nevertheless, consistent evidence indicating a higher incidence of CH in families with other affected members compared with the general population suggests a predisposing genetic risk which remains unidentified. To date, there has been only one hypothesis-free genetic familial study that examined linkage in five Danish pedigrees. It did not produce a significant logarithm of the odds (LOD) score but showed a suggestion of linkage at loci on chromosomes 2, 8 and 9. Unfortunately, this was not replicated when extended to the entire cohort (31).

A review of pedigrees revealed a pattern most consistent with an autosomal dominant mode of inheritance in the majority of cases (40/48 families). Some families exhibited evidence of reduced penetrance; however this could also be reflective of a cumulative effect of a number of disease-associated variants. The remaining pedigrees, more consistent with an autosomal recessive inheritance pattern, were small in size and may also represent a dominant mode of inheritance with reduced penetrance. The apparent incongruity in inheritance patterns across families correlates with previous familial studies in CH (19). Explanations for this includes reduced penetrance, loci heterogeneity, genetic pleiotropy, and the presence of modifying variants, which augment or attenuate the effect of inherited pathogenic mutations.

In contrast to previous studies, we did not identify a higher proportion of females amongst familial cases (32). This is possibly reflective of the evolving demographics of CH in more recent years, with a considerable increase in the diagnosis of female patients. In earlier studies, it is possible that an underrepresentation of females provided insufficient power for gender segregation analysis (7). A younger age of onset has been observed in familial CH, particularly in female patients (33). The possibility of anticipation has previously been postulated, however, this would require large pedigrees exhibiting a decreasing age of onset across successive generations (19). This difference may indicate an earlier recognition of familial CH in patients acquainted with the condition in their relatives. It is also possible that variability in age of onset is determined by distinct genetic variants inherited within families. We did not identify an earlier age of onset on multivariate analysis. Larger, well-designed studies are required to establish this definitively.

Autonomic symptoms were more prominent in the familial group on univariate analysis. The autonomic symptom of nasal blockage remained significant on multivariate analysis. Larger studies are required to examine this association however it raises interest in genetic candidates associated with pain conditions with prominent dysautonomia such as *SCN9A* [OMIM: 603415] in paroxysmal extreme pain disorder (PEPD) and *SCN11A* [OMIM: 604385] in familial episodic pain syndrome (FEPS3) (34,35).

Patients with familial CH were also more likely to have a concomitant SUNCT, a TAC with an estimated prevalence of 1.2/100,000 people (36). We see a higher volume of such patients with this condition at our tertiary referral headache clinic, but considering the rarity of this disorder and CH, their co-existence in familial cases is unlikely to be coincidental. Also, although SUNCT shares clinical features with CH, these conditions are clearly phenotypically distinct. SUNCT presents with considerably shorter attacks (1–600 seconds) and responds to different treatments including intravenous lidocaine and prophylactic lamotrigine (37).

Patients with one headache disorder are at higher risk of developing another form of headache, possibly reflecting a predisposition to primary headache due to central sensitization of the pain matrix. The co-occurrence of more than one TAC in a patient is unusual, but has been reported previously (38). It is plausible that this overlap implies a shared pathophysiological mechanism between both syndromes leading to the activation of trigeminovascular system. Furthermore, activation of the posterior hypothalamus in functional imaging is evident in both CH and SUNCT, possibly representing a derangement in the regulation of hypothalamic neurotransmitters common to both conditions (39). The higher proportion of familial cases with SUNCT and CH suggests that genetic variation may be a common denominator driving this pathophysiological pathway, thus predisposing to both syndromes. SUNCT does not respond to oxygen. Interestingly, our univariate analysis indicated that patients with familial CH had a poorer response to high-flow oxygen which was lost on multivariate analysis. This finding may be a directly influenced by the co-occurrence of SUNCT, whereby patients do not derive improvement from oxygen with every headache attack.

In conclusion, a confirmed family history in 7.44% of this cohort further supports the role of heredity in the pathophysiology of CH. Additionally, we found that nasal blockage and concurrent SUNCT is distinctly more common in familial cases, potentially suggesting that genetic variation may influence phenotype. Specifically, we did not replicate the findings of a younger age of onset or difference in the gender ratio in familial cases as previously demonstrated. A limitation to this study is the small cohort size due to the rarity of familial CH. It is also limited by potential recall bias, especially in relatives affected with CH. Also, our dataset contained some missing values requiring imputation, however the random forest imputation technique employed does not change the structure of the data. The potential disadvantage of using the ROSE algorithm is losing potential useful information on the dataset by shrinking the existing one. We observed that the results did not change significantly after implementation of data imputation and the subsequent balancing of data. The LASSO algorithm, as with other machine learning techniques, is blind and therefore results should be interpreted in this context. Finally, this study was conducted in a tertiary care setting and replication of our findings in a secondary care setting is required. Nonetheless, these results add to evidence indicating that genetic variation likely contributes to the development of CH. Further studies investigating the genetic architecture of CH are required to understand the genotype-phenotype correlation and its potential impact on mechanistic studies and therapeutic intervention.

## Key findings

- A positive family history was confirmed in 7.44% of patients.
- Autosomal dominant with or without reduced penetrance was the most frequently observed modes of inheritance.
- Familial CH is associated with prominent nasal blockage and concomitant SUNCT.
- This study was unable to confirm an earlier age of onset or higher rate of females in familial CH.

## Supplemental Material

sj-pdf-1-cep-10.1177_03331024221076478 - Supplemental material for The clinical characteristics of familial cluster headache Click here for additional data file. Supplemental material, sj-pdf-1-cep-10.1177_03331024221076478 for The clinical characteristics of familial cluster headache by Emer O’Connor, Elham Nikram, Lou Grangeon, Daisuke Danno, Henry Houlden and Manjit Matharu in Cephalalgia

sj-pdf-2-cep-10.1177_03331024221076478 - Supplemental material for The clinical characteristics of familial cluster headache Click here for additional data file. Supplemental material, sj-pdf-2-cep-10.1177_03331024221076478 for The clinical characteristics of familial cluster headache by Emer O’Connor, Elham Nikram, Lou Grangeon, Daisuke Danno, Henry Houlden and Manjit Matharu in Cephalalgia
