# Supplementary material for: The clinical characteristics of familial cluster headache
Source: Cephalalgia. 2022 Feb 15;42(8):715–21. doi: 10.1177/03331024221076478 (PMC9218408; doi:10.1177/03331024221076478)
Supplement: sj-pdf-1-cep-10.1177_03331024221076478 - Supplemental material for The clinical characteristics of familial cluster headache [file sj-pdf-1-cep-10.1177_03331024221076478.pdf]

Supplementary Table 1: Treatment Response in Familial Cluster Headache																		
ACUTE TREATMENTS					PROPHYLACTIC TREATMENTS													
**ID**	**O2**	**O2 SR**	**SUMA**	**SUMA SR**	**VERA**	**VERA SR**	**LIT**	**LIT SR**	**METH**	**METH SR**	**MEL**	**MEL SR**	**TOP**	**TOP SR**	**GABA**	**GABA SR**	**NM**	**NM SR**
**p1**	Y	Y	N	-	N	-	N	-	N	-	N	-	N	-	N	-	ONS	Y
**p2**	Y	Y	Y	Y	Y	Y	N	-	N	-	N	-	N	-	N	-		
**p3**	Y	Y	Y	N	Y	N	N	-	N	-	N	-	Y	Y	Y	N		
**p4**	Y	N	NA	NA	NA	NA	NA	NA	NA	NA	NA	NA	NA	NA	NA	NA	NA	NA
**p5**	Y	Y	Y	Y	Y	N	Y	N	N	-	Y	Y	Y	N	Y	N	ONS	Y
**p6**	N	-	N	-	N	-	N	-	N	-	N	-	N	-	N	-		
**p7**	N	NA	N	NA	NA	NA	NA	NA	NA	NA	NA	NA	N	-	N	-		
**p8**	Y	Y	N	-	Y	N	Y	Y	N	-	N	-	N	-	N	-		
**p9**	Y	Y	Y	Y	Y	Y	Y	N	Y	N	Y	N	N	-	N	-		
**p10**	Y	N	Y	Y	Y	N	Y	N	Y	N	Y	N	Y	N	Y	N		
**p11**	Y	Y	N	-	N	-	N	-	N	-	N	-	N	-	N	-	ONS	Y
**p12**	Y	Y	Y	Y	Y	N	N	-	N	-	N	-	Y	Y	N	-		
**p13**	Y	N	Y	N	Y	N	Y	N	N	-	Y	N	Y	N	Y	N		
**p14**	Y	Y	Y	Y	Y	N	Y	N	Y	N	N	-	Y	N	Y	N		
**p15**	Y	N	Y	Y	Y	N	Y	N	Y	N	Y	N	N	-	Y	N		

**p16**	Y	Y	N	-	Y	Y	N	-	N	-	N	-	Y	N	Y	N		
**p17**	Y	Y	Y	Y	Y	N	Y	N	N	-	Y	Y	Y	N	Y	N		
**p18**	N	-	Y	Y	N	-	N	-	N	-	N	-	N	-	N	-		
**p19**	N	-	NA	NA	NA	NA	NA	NA	NA	NA	NA	NA	NA	NA	NA	NA	NA	NA
**p20**	Y	N	Y	Y	Y	Y	N	-	Y	Y	N	-	N	-	N	-		
**p21**	N	-	NA	NA	NA	NA	NA	NA	NA	NA	NA	NA	NA	NA	NA	NA		
**p22**	Y	Y	Y	Y	Y	N	N	-	N	-	N	-	Y	Y	N	-		
**p23**	Y	Y	Y	Y	Y	N	Y	N	Y	N	Y	N	N	-	Y	N		
**p24**	N	-	N	-	Y	N	Y	N	Y	N	N	-	N	-	Y	N		
**p25**	Y	Y	Y	N	Y	N	Y	N	Y	N	Y	N	N	-	Y	N		
**p26**	N	-	Y	Y	N	-	N	-	N	-	N	-	N	-	N	-		
**p27**	Y	Y	Y	Y	Y	Y	Y	Y	N	-	N	-	N	-	N	-		
**p28**	Y	Y	Y	N	Y	N	N	-	N	-	Y	Y	N	-	N	-		
**p29**	Y	Y	Y	Y	N	-	N	-	N	-	N	-	N	-	N	-	ONS	Y
**p30**	Y	Y	Y	Y	Y	Y	Y	N	Y	N	Y	N	N	-	N	-	ONS<br>DBS	N<br>Y
**p31**	N	-	N	-	N	-	N	-	N	-	N	-	N	-	Y	N	ONS	N
**p32**	Y	Y	Y	Y	Y	N	Y	N	Y	Y	Y	N	Y	N	N	-		
**p33**	Y	Y	Y	Y	Y	N	N	-	Y	N	Y	N	Y	N	Y	Y		
**p34**	Y	N	NA	NA	NA	NA	NA	NA	NA	NA	NA	NA	NA	NA	NA	NA		

**p35**	Y	Y	Y	Y	Y	Y	N	-	N	-	N	-	N	-	Y	N		
**p36**	Y	Y	Y	Y	Y	N	Y	N	Y	Y	Y	N	Y	N	N			
**p37**	N	-	Y	Y	Y	N	Y	N	N	-	N	-	N	-	Y	N		
**p38**	N	-	Y	Y	Y	N	Y	Y	N	-	N	-	Y	N	N	-		
**p39**	Y	Y	Y	Y	Y	N	Y	N	N	-	Y	Y	Y	N	Y	N		
**p40**	N	-	N	-	N	-	N	-	N	-	N	-	N	-	Y	N		
**p41**	Y	N	Y	Y	Y	Y	N	-	Y	N	N	-	Y	N	N	-		
**p42**	Y	Y	Y	Y	Y	Y	Y	Y	Y	N	N	-	Y	Y	Y	N		
**p43**	Y	Y	Y	N	Y	Y	N	-	N	-	N	-	N	-	N	-		
**p44**	Y	Y	Y	N	Y	N	Y	Y	Y	N	Y	N	Y	N	Y	N		
**p45**	N	-	Y	Y	N	-	N	-	N	-	N	-	N	-	N	-	ONS	Y
**p46**	Y	N	NA	NA	N	-	Y	Y	Y	N	N	-	Y	N	Y	N		
**p47**	Y	Y	N	N	Y	N	Y	N	N	-	N	-	Y	N	N	-		
**p48**	Y	N	NA	NA	NA	NA	NA	NA	NA	NA	NA	NA	NA	NA	NA	NA		

Abbreviations: DBS = Deep Brain Stimulation, GABA = Gabapentin, LIT = Lithium, MEL = Melatonin, METH = Methysergide, N= No, NA= Not Available, NM= Neuromodulation, O2= Oxygen, ONS = Occipital Nerve Stimulator, SR = Satisfactory response (see supplementary table 1), SUMA = Sumatriptan, TOP = Topiramate, VERA = Verapamil, Y= Yes.

Supplementary Table 2: Modified intractable CH definition (on the basis of Goadsby et al, 2009)

Adequate trial performed

- Appropriate dose : Decision left to the clinical physician
- Appropriate length of time:
  At least 1 month for melatonin trial
  At least 3 months for all other preventive therapeutics

Failed trial

- Unsatisfactory response:
  Less than 50% reduction in mean attack frequency for preventive treatment
  Less than 50% reduction in pain at least 50% of the time for acute treatment
- Side-effects requiring cessation of treatment
- Contraindications to use

Intractable to acute treatment

- Failure within 15 minutes of subcutaneous sumatriptan injection
- And/or failure within 30minutes of high dose and flow rate oxygen

Intractable to preventive treatment:

Failure of at least 4 classes among:

Verapamil

Lithium

Topiramate

Gabapentin

Methysergide

Melatonin

Supplementary Table 3: Baseline demographics and clinical characteristics of cohorts, with corresponding univariate analysis following imputation and re-balancing of cohorts.									
**Cohorts**	**Demographics and Clinical Characteristics**			**Imputed Cohorts**			**Imputed and re-balanced cohorts & Univariate Analysis**		
	**FCH** (n=48)(%)	**SCH** (n=597) (%)	*P*	**FCH Imp** (n=48)(%)	**SCH Imp** (n=597)(%)	*P*	**FCH ROSE sample** (n=313)(%)	**SCH ROSE sample** (n=332)(%)	*P*
**Age**	48.91+/-12.05	49.49+/-12.46	0.89	48.91+/-12.05	49.53+/-12.37	0.87	47.93+/-12.91	49.94+/-13.56	0.09
**Gender M:F**	35 : 13	421:176	0.85	35:13	421:176	0.85	225:88	237:95	0.95
**Age of onset**	28.48+/-13.09	31.29+/-13.13	0.12	28.61+/-12.99	31.29+/-13.07	0.13	27.53+/-14.25	31.80+/-14.27	**<0.001**
**Chronic**	21 (43.75)	285 (47.73)	0.70	21 (43.75)	285 (47.73)	0.70	137 (43.76)	166 (50.00)	0.13
**Site**									
**Orbital**	33 (68.8)	419 (70.18)	0.98	34 (70.8)	422 (70.68)	1	228 (72.84)	227 (68.37)	0.24
**Frontal**	14 (29.1)	203 (34)	0.66	14 (29.1)	210 (35.1)	0.49	86 (27.47)	119 (35.84)	**0.02**
**Temporal**	22 (45.8)	304 (50.9)	0.60	22 (45.8)	313 (52.4)	0.46	147 (46.96)	171 (51.50)	0.28

**Parietal**	7 (14.5)	106 (17.7)	0.72	7 (14.5)	116 (19.43)	0.52	48 (15.33)	70 (21.08)	0.07
**Occipital**	9 (18.75)	122(20.43)	0.93	9 (18.75)	132 (22.1)	0.75	42 (13.41)	82 (24.69)	**<0.001**
**Cheek**	14 (29.2)	136 (22.8)	0.39	15 (31.2)	142 (23.7)	0.32	90 (28.75)	68 (20.48)	**0.018**
**Teeth**	4 (8.3)	59 (9.88)	0.83	5 (10.41)	67 (11.22)	0.91	33 (10.54)	42 (12.65)	0.47
**Ear**	4 (8.3)	59 (9.88)	0.83	4 (8.3)	67 (11.22)	0.71	24 (7.66)	35 (10.54)	0.25
**Autonomics**									
**Absence of Autonomics**	1 (2.1)	13 (2.2)	1	1 (2.1)	25 (4.8)	0.71	3 (0.95)	13 (3.91)	0.62
**Ptosis**	28 (58.3)	345 (57.8)	0.69	32 (66.6)	383 (64.15)	0.84	207 (66.13)	211 (63.55)	0.54
**Eyelid oedema**	21 (43.8)	213 (35.7)	0.14	25 (52.08)	253 (42.37)	0.24	176 (56.23)	128 (38.55)	**<0.001**
**Conjunctival Injection**	34 (70.8)	401 (67.2)	0.29	39 (81.25)	447 (74.87)	0.41	270 (86.26)	247 (74.39)	**<0.001**
**Miosis**	1 (2.1)	22 (3.7)	0.60	7 (14.58)	57 (9.54)	0.38	49 (15.65)	35 (10.54)	0.07

**Lacrimation**	39 (81.25)	472 (79.1)	0.22	43 (89.58)	511 (85.59)	0.58	283 (90.41)	281 (84.63)	**0.03**
**Nasal blockage**	36 (75)	350 (58.6)	**0.004**	43 (89.58)	404 (67.67)	**0.002**	278 (88.81)	218 (65.66)	**<0.001**
**Rhinorrhoea**	30 (62.5)	364 (60.9)	0.41	37 (77.08)	404 (67.67)	0.23	245 (78.27)	244 (73.49)	0.18
**Facial Sweating**	23 (47.9)	295 (49.4)	0.85	30 (62.5)	337 (56.44)	0.50	201 (64.21)	175 (52.71)	**0.003**
**Flushing**	19 (39.6)	231 (38.7)	0.68	22 (45.83)	249 (41.7)	0.68	142 (45.36)	123 (37.04)	**0.03**
**Aural Fullness**	8 (16.7)	105 (17.6)	1	14 (29.16)	122 (20.43)	0.35	88 (28.11)	71 (21.38)	0.06
**Agitation**	40 (83.3)	498 (83.4)	0.82	44 (91.66)	517 (86.59)	0.43	288 (92.01)	287 (86.44)	**0.03**
**Frequency / Duration of attacks**									
**Average Attacks per day**	3.08+/-2.18	2.99+/-2.18	0.61	3.08+/-2.18	2.99+/-2.18	0.63	3.11+/-2.56	2.96+/-2.29	0.50
**Average Duration (mins)**	67.22+/-43.56	94.97+/-132.36	**0.01**	67.22+/-43	95+/-131.50	**0.01**	70.35+/-49.42	91.49+/-90.86	**<0.001**
**Associated Headaches**									
**Migraine**	15 (31.3)	180 (30.2)	1	15 (31.3)	181 (30.31)	1	85 (27.15)	97	0.62

								(29.21)	
**TACS**	3 (6.25)	20 (3.3)	0.24	3 (6.25)	21 (3.51)	0.41	22 (7.02)	8 (2.40)	**<0.009**
**Treatment Response**									
**Response to Oxygen**	9 (18.7)	84 (14.1)	0.97	11 (22.91)	192 (32.16)	0.24	65 (20.76)	105 (31.62)	**0.002**
**Response to sc Sumatriptan**	6 (12.5)	29 (4.8)	0.10	13 (27.08)	141 (23.61)	0.71	85 (27.15)	80 (24.09)	0.42
**Intractable to preventative treatment**	24 (50)	244 (40.8)	0.47	35 (72.91)	399 (66.83)	0.48	227 (72.52)	217 (65.36)	0.06

Abbreviations: CH: Cluster Headache, M: male, F: female, FCH : Familial Cluster Headache, Imp: Imputed, sc: subcutaneous, TACS: Trigeminal Autonomic Cephalalgia.
