# Supplementary material for: The clinical characteristics of familial cluster headache
Source: Cephalalgia. 2022 Feb 15;42(8):715–21. doi: 10.1177/03331024221076478 (PMC9218408; doi:10.1177/03331024221076478)
Supplement: sj-pdf-2-cep-10.1177_03331024221076478 - Supplemental material for The clinical characteristics of familial cluster headache [file sj-pdf-2-cep-10.1177_03331024221076478.pdf]

Supplementary Figure 1

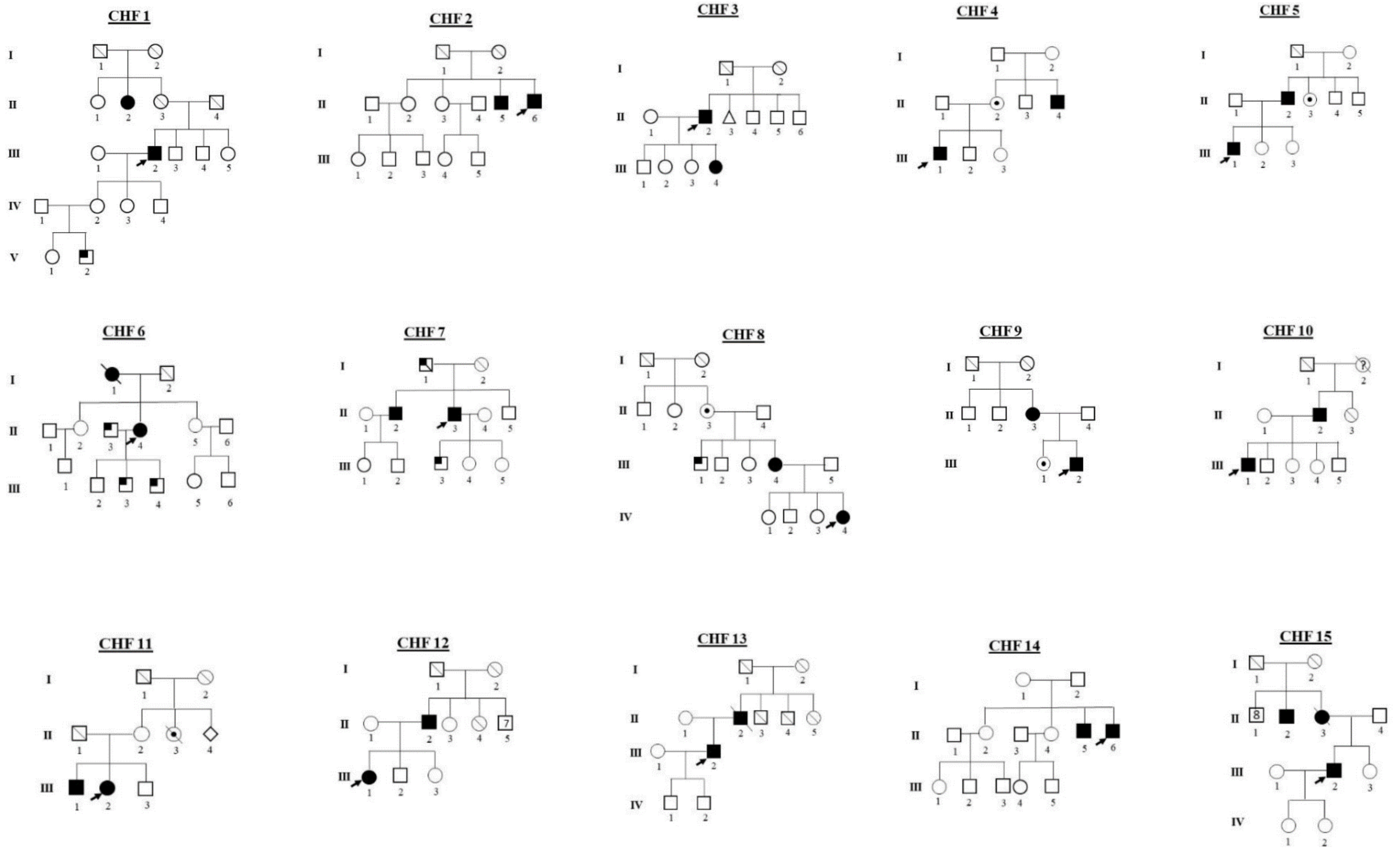

CHF 16

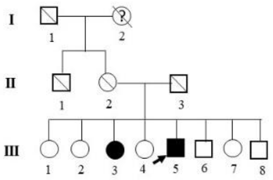

CHF 17

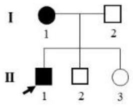

CHF 18

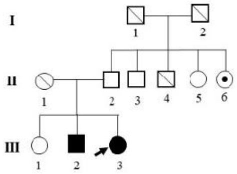

CHF 19

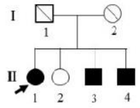

CHF 20

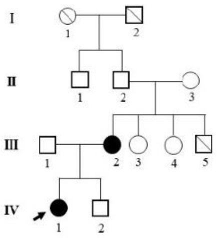

CHF 21

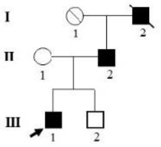

CHF 22

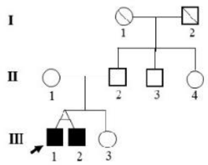

CHF 23

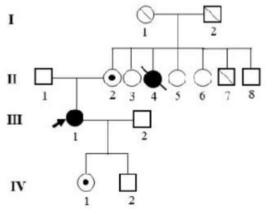

CHF 24

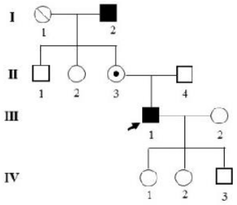

CHF 25

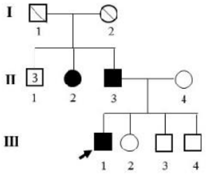

CHF 26

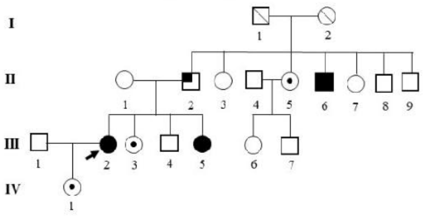

CHF 27

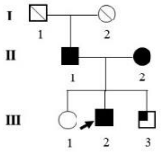

CHF 28

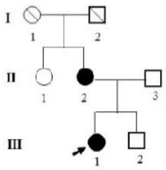

CHF 29

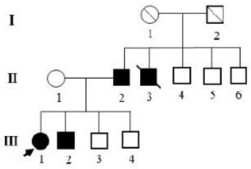

CHF 30

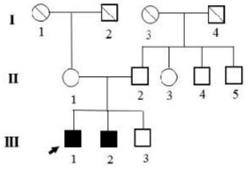

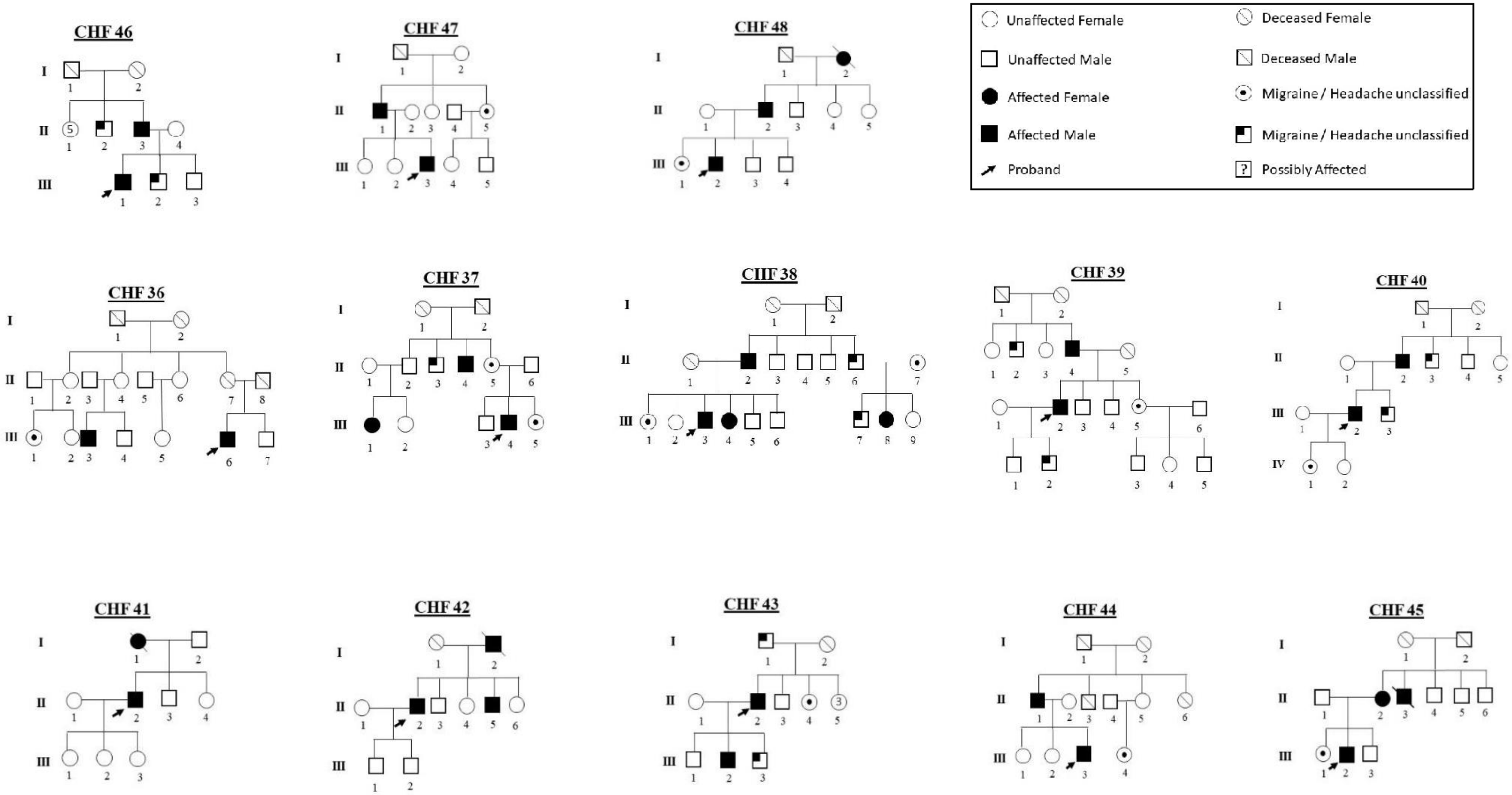
CHF 46
CHF 47
CHF 48
Unaffected Female
Deceased Female
Unaffected Male
Deceased Male
Affected Female
Migraine / Headache unclassified
Affected Male
Migraine / Headache unclassified
Proband
Possibly Affected
CHF 36
CHF 37
CHF 38
CHF 39
CHF 40
CHF 41
CHF 42
CHF 43
CHF 44
CHF 45

Supplementary Figure 1: Pedigrees for families 1-48. Probands are indicated with an arrow and individuals affected with cluster headache are shown in black.
